# Identification of plasmid IncQ1 and NTE_KPC_-IId harboring *bla*_KPC-2_ in isolates from *Klebsiella pneumoniae* infections in patients from Recife-PE, Brazil

**DOI:** 10.1590/0037-8682-0526-2019

**Published:** 2020-06-22

**Authors:** Giselle Jucá de Lima, Alexsandra Maria Lima Scavuzzi, Elizabeth Maria Bispo Beltrão, Elza Ferreira Firmo, Érica Maria de Oliveira, Sibele Ribeiro de Oliveira, Antônio Mauro Rezende, Ana Catarina de Souza Lopes

**Affiliations:** 1 Universidade Federal de Pernambuco, Departamento de Medicina Tropical, Recife, PE, Brasil.; 2 Centro Universitário Tabosa de Almeida, Asces-UNITA, Caruaru, PE, Brasil.; 3 Instituto Aggeu Magalhães (IAM-Fiocruz), Recife, PE, Brasil.

**Keywords:** Klebsiella pnemoniae, IncQ1, *bla*_KPC-2_, NTE_KPC-_IId

## Abstract

**INTRODUCTION:**

This study investigated the genetic environment of *bla*_KPC-2_ in *Klebsiella pnemoniae* multi-drug resistant clinical isolates.

**METHODS:**

Four carbapenemase gene isolates resistant to carbapenems, collected from infected patients from two hospitals in Brazil, were investigated using polymerase chain reaction and plasmid DNA sequencing.

**RESULTS:**

The *bla*_KPC-2_ gene was located between IS*Kpn6* and a resolvase *tnpR* in the non-Tn*4401* element (NTE_KPC-_IId). It was detected on a plasmid belonging to the IncQ1 group.

**CONCLUSIONS:**

To our knowledge, this is the first report of the presence of the *bla*_KPC-2_ gene in the NTE_KPC_-IId element carried by plasmid IncQ1 from infections in Brazil.

*Klebsiella pneumoniae* is one of the pathogens responsible for healthcare-associated infections (HAIs). Infections can, depending on the anatomic site affected and the patient’s immune status,lead to a range of adverse clinical outcomes, including death, as this gram-negative bacterium carries several antibiotic resistance and virulence genes^1^. 

The most relevant antibiotic-resistant genes in this bacterial species are those that encode carbapenemases, (KPC). *K. pneumoniae* isolates from different countries, including Brazil, have been found to contain the KPC encoding gene *bla*_KPC-2_. This gene has been found to be located on plasmids of different sizes and nucleotide sequences and belong to different incompatibility groups (Incs), the most prevalent being IncL/M, IncFII and IncN^2^ ^,^ ^3^. 

Nicoletti et al. (2015)^4^ identified the IncQ plasmid in *K. pneumoniae* carrying carbapenemase BKC-1 from São Paulo, Brazil. IncQ1 is a stable and mobilizable plasmid that can be transferred among a wide range of gram negative bacteria through conjugative plasmids present in the same bacterial cell, which facilitates its transmission in a hospital environment. There are few studies in Brazil that characterize the plasmid Incs of *K. pneumoniae*, mainly because some of these are non-typeable plasmids, such as the ones studied by Pereira et al (2013)^5^. 

These plasmids may also harbor different isoforms of the Tn*4401* transposon. Nine variants of Tn*4401* (a-i) have been described^6^, of which variants "a" and "b" are the most common. Non-Tn*4401* (NTE_-KPC_)^7^ ^,^ ^8^ elements that can carry *bla*_KPC-2_ have also been described, including those recently detected in two colonization isolates in Brazil^8^.

The aim of this study was to investigate the genetic environment of the *bla*_KPC-2_ gene from clinical isolates of *K. pneumoniae* resistant to carbapenems, thus helping to understand the dissemination of carbapenem resistance. This may help develop new strategies to prevent the spread of these resistance genes in the hospital environment.

Four multi-drug resistant (MDR) clinical isolates of *K. pneumoniae* (K3R2, K4R2, K6R2, and K1E) were selected, following isolation, from infections (peritoneal fluid, blood cultures, and cerebrospinal fluid) in patients from two hospitals in Recife-PE, Brazil in 2016. The isolates were identified biochemically using the automated VITEK® 2 method. The isolates were stored in 20% glycerol at -70°C. For the analyses, the isolates were cultured in BHI (Brain Heart Infusion) broth or Luria Bertani (LB) for 18 hours at 37°C.

Susceptibility to different classes of antimicrobials was assessed using the automated Bactec 9120 (Phoenix BD) system. The antimicrobials tested were amikacin, ampicillin, ampicillin/sulbactam, cefalotin, ceftazidime, cefepime, cefoxitin, ciprofloxacin, ceftriaxone, cefuroxime, colistin, gentamycin, ertapenem, imipenem, meropenem, and tigecycline. The interpretation was performed according to the criteria of the Clinical and Laboratory Standards Institute (CLSI 2017)^9^. 

The genomic DNA of the isolates was extracted by the Wizard Genomic DNA purification kit (Promega- Brazil). The *bla*_KPC-2_ gene was investigated usingpolymerase chain reaction (PCR) using previously described primers and amplification conditions^2^. Negative and positive controls were included in each PCR. The amplified products were electrophoresed in 1% agarose gel under constant voltage of 100 V in 0.5 × TBE buffer (Tris-base, boric acid, and ethylenediamine tetra-acetic acid, EDTA- pH8,0).

The enterobacterial repetitive intergenic consensus (ERIC)-PCR method was used to determine the clonal relationship of the isolates using previously described primers and amplification conditions^2^. The amplified products were electrophoresed in 1.5% agarose gel under a constant voltage of 100V in 0.5 × TBE buffer. 

Plasmid DNA was extracted using the Plasmid Mini Kit (Qiagen), was quantified using the NanoDrop spectrophotometer and Qubit fluorometric platform (ThermoFisher Scientific). The libraries were built using the Nextera XT Library Preparation (Illumina) and were quantified via real-time PCR using the Library Quantification kit - Illumina/Universal (Kapa Biosystems). Sequencing was performed using MiSeq equipment (Illumina) with the MiSeq 500-cycle cartridge Nano kit V2 (Illumina). The data were processed using the Trimmomatic tool^10^, and *de-novo* assemblies were performed using the Velvet tool^11^. The annotated plasmid DNA sequences were visualized using Artemis Sanger software^12^. 

The IncQ plasmid was identified using PCR with the primers and the amplification conditions described by Götz et al. (1996)^13^. The PCR products were electrophoresed on 1.0% agarose gel in TBE buffer. The IncQ plasmid was also identified using *in silico* PCR; bioinformatics tools used included sequence manipulation suite (SMS) (http://www.bioinformatics.org/sms2/index.html) and primer-basic local alignment search tool (BLAST), using the primers for determination of all different plasmid incompatibility groups, as defined by Carattoli et al (2005)^14^. 

The isolates of *K. pneumoniae* were MDR, with resistance to β-lactams, and especially to carbapenems (Table 1), and they were suspected of being producers of KPC. Using PCR, the presence of the *bla*_KPC-2_ gene in the four *K. pneumoniae* isolates analyzed was confirmed. The ERIC-PCR genotyping test showed that all the isolates presented distinct clonal profiles, with a maximum of 40% similarity, and therefore they did not present a clonal relationship (Table 1).The plasmid DNA was sequenced to a depth of approximately 238 times. The analysis of the plasmid DNA sequences from all isolates using the Resfinder and GenBank databases confirmed the presence of the *bla*_KPC-2_ (882 bp) antibiotic-resistance gene. The gene was identified with 100% similarity when compared with a sequence deposited in GenBank (CP023186.1). The *bla*_KPC-2_ gene was observed in similar genetic locations of all isolates and was inserted between the ΔIS*Kpn6* insert sequence and a resolvase *tnpR;* between 402-558 bp upstream of the *bla*_KPC-2_ gene, a truncated *bla*_TEM_ gene was an evidence that *bla*_KPC-2_ was inserted into a non-Tn*4401* (variant NTE_KPC_-IId) (Figure 1). We found deletions in *tnpA* and a total deletion of the ISK*pn7* insert sequence (Figure 1). 

**TABLE 1:** Source of isolation, origin, ERIC-PCR profile, and resistance profile of clinical MDR isolates of *K. pneumoniae*, Recife-PE, Brazil, 2016.

Isolates	Origin	ERIC-PCR profile	Resistance profile
K3R2	Peritoneal fluid	P1	AMP, APS, CFL, CFZ, CFP, CXM, CAZ, CRO, CFX, CIP, ERT, IPM, MER
K4R2	Blood culture	P2	AMP, APS, CFL, CFZ, CFP, CXM, CAZ, CRO, CFX, CIP, ERT, IPM, MER
K6R2	Cerebrospinal fluid	P3	AMP, APS, CFL, CFZ, CFP, CXM, CAZ, CRO, CFX, CIP, ERT, IPM, MER
K1E	Blood culture	P5	AMP, APS, CFL, CFZ, CFP, CAZ, CRO, CFX, CIP, ERT, GEN, IPM, MER

**K:** *Klebsiella pneumoniae*; **R2:** public hospital; **E:** private hospital; **P:** profile; **AMP:** ampicillin; **APS:** ampicillin/sulbactam; **CFL:** cefalotin; **CFZ:** cefazolin; **CFP:** cefepime; **CFX:** cefoxitin, **CAZ:** ceftazidime; **CRO:** ceftriaxone; **CXM:** cefuroxime; **CIP:** ciprofloxacin; **ERT:** ertapenem; **GEN:** gentamycin, **IPM:** imipenem; **MER:** meropenem.

**FIGURE 1:**
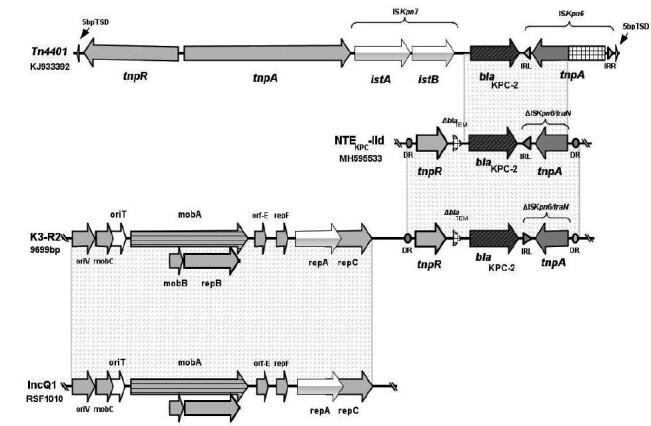
Comparison among Tn*4401*, non-Tn*4401* (NTE KPC -IId), and IncQ1 carrying the *bla*_KPC-2_ gene in *Klebsiella pneumoniae*. Tn*4401* with *bla*_KPC-2_ gene between ISKpn6 and ISKpn7; NTEKPC-IId with deletion of the ISK*pn7* and truncated *bla*_TEM_ gene; K3-R2 isolate with *bla*_KPC-2_ gene between truncated *bla*_TEM_ gene and ΔIS*Kpn6* as well as mobility and replication genes encoding the plasmid IncQ1. Protein-coding sequences are represented by the arrows and labeled with gene name. Vertical lines represent gaps schematizing the termination of one contig and the beginning of another contig in the isolate. Gray fill represents homologous shared regions. The direct repeat sequence of NTE KPC -IId is represented by a circle.

In the same consensus sequence where the non-Tn*4401* was located, plasmid mobility proteins (*mobA, mobB,* and *mobC*) and replication proteins (*repA, repB,* and *repC*) were found with a 100% similarity to the reference pool of the IncQ1 RSF1010 (M28829.1).

The *oriV, oriT,* and *repB* genes were also found inserted into the same consensus sequence of the replication and mobilization genes as the *bla*_KPC-2_ gene. Thus, this result suggests that all the isolates have the genes encoding the plasmid IncQ1 and the *bla*_KPC-2_ gene in the same consensus sequence (Figure 1 and Table 2).

PlasmidFinder confirmed the presence of a plasmid belonging to the variant incompatibility group IncQ1. The *in silico* PCR with all the isolates, tested positive for the IncQ1 replicons (*oriV*-436bp, *oriT*-191bp, and *repB*-1160pb). Comparative analysis among the four *K. pneumoniae* isolates of this study and reference sequences for the IncQ1 and IncQ-like plasmids deposited in GenBank showed 98% to 100% similarity to the *oriV* gene (M21475.1), to the *oriT* gene (X04830.1), and to the *repB* gene (M28829.1). 

The PCR for the IncQ1 replicons also confirmed this result, with the *repB, oriV*, and *oriT* genes of the plasmid IncQ1 amplified in all isolates analyzed. In addition to IncQ1, the isolates also presented other plasmids, but these were not typable given the total size of the DNA sequence (Table 2).

**TABLE 2:** Plasmid sequence characteristics of *K. pneumoniae* isolates and comparative analysis with plasmid RSF1010-IncQ (accession number: M28829.1).

Isolates	K3R2	K4R2	K6R2	K1E	IncQ (M28829.1)
**Reference**	This study	This study	This study	This study	Scholz et al. (1989)
Size of the sequenced plasmid DNA	27,508 bp	27,325 bp	59,776 bp	84,800 bp	8,684 bp
GC	57.95%	58.13%	55.35%	53.84%	61%
CDS	31	31	70	94	40
Inc	IncQ1 and NTP	IncQ1 and NTP	IncQ1 and NTP	IncQ1 and NTP	IncQ1 and NTP
Resistance genes	*bla*_*KPC-2*_	*bla*_*KPC-2*_	*bla*_*KPC-2*_	*bla*_*KPC-2*_	*str sul*
Mobility and replication	*mobA*	*mobA*	*mobA*	*mobA*	*mobA*
Proteins	*mobB*	*mobB*	*mobB*	*mobB*	*mobB*
	*mobC*	*mobC*	*mobC*	*mobC*	*mobC*
	*repA*	*repA*	*repA*	*repA*	*repA*
	*repB*	*repB*	*repB*	*repB*	*repB*
	*repC*	*repC*	*repC*	*repC*	*repC*
	*repF*	*repF*	*repF*	*repF*	*repF*

**K:** *Klebsiella pneumoniae*; **R2:** public hospital; **E:** private hospital; **GC:** guanine and cytosine; **bp:** base pairs; **CDS:** coding sequence; **Inc:** plasmid incompatibility group; **KPC:** *Klebsiella pneumoniae* carbapenemase; ***str:*** streptomycin; ***sul:*** sulfonamide; **mob:** mobility protein; **rep:** replication proteins; **NTP:** not typable.

Antimicrobial resistance genes are spread among enterobacteria due to the horizontal transfer of mobile genetic elements. The *bla*_KPC-2_ gene is found associated with several different plasmids^1^ ^,^ ^7^. However, little was known about the plasmid genetic environment of this gene in clinical isolates of *K. pneumoniae* in Brazil, and especially in Recife-PE, where the first reports of KPC in Brazil came from.

The *bla*_KPC-2_ gene is often found inserted into transposon Tn*440*, which has different isoforms, but it has also been found in a non-Tn*4401* mobile element (NTE_KPC_) in China, Argentina, Brazil, and Russia^7^ ^,^ ^15^ ^,^ ^16^. NTE_KPC_ has been separated into three groups based on the absence or presence of the *bla*_TEM_ gene, where the second group, NTE_KPC_-II, includes the variant with a truncated *bla*_TEM_ gene^15^ ^,^ ^16^.

The non-Tn*4401* variant of the present study resembles the NTE_KPC-_IId variant (Figure 1). These findings corroborate the results obtained by our research group with *Klebsiella aerogenes* in Recife-PE, Brazil, which had 100% similarity with a sequence deposited in GenBank (MG786907, MH000708).

IncQ and IncQ-like plasmids have been found in different bacterial species such as *Escherichia coli, Salmonella typhimurium, Salmonella enterica serovar, Pseudomonas aeruginosa,* and *Enterobacter cloacae* from locations in Canada, Italy, the United Kingdom, and Germany^17^. 

This report demonstrates the presence of the *bla*_KPC-2_ gene in the non-Tn4401 element (NTE_KPC-_IId), which is carried by small, mobilizable, and promiscuous plasmids of the type IncQ1, in four clinical MDR isolates of infection by *K. pneumoniae* in Northeast Brazil. This data indicates that this type of plasmid may have been responsible for spreading the *bla*_KPC-2_ gene among *K. pneumoniae* in patients from hospitals in Recife, Brazil. 

The study by Pereira et al. (2013)^5^ used *K. pneumoniae* isolates from Recife-Pernambuco, Brazil, but it was not possible to type the plasmids. Cerdeira et al. (2019)^7^ found the *bla*_KPC-2_ gene in the NTE_KPC-_IId gene carried by IncQ1plasmids in two colonization isolates of *K. pneumoniae* in Brazil (uninformed locality).

Collectively, these results reveal the dynamics of the genetic environment of the *bla*_KPC-2_ gene and emphasize the continuous recombination and evolution of plasmids and transposons. This may make the spread of different resistance genes in *K. pneumoniae* isolates more likely, introducing additional difficulties to the development of possible measures to control the spread of this form of bacterial resistance.
